# Solid-Phase Extraction of Active Compounds from Natural Products by Molecularly Imprinted Polymers: Synthesis and Extraction Parameters

**DOI:** 10.3390/polym13213780

**Published:** 2021-10-31

**Authors:** Sazlinda Kamaruzaman, Najihah Mohammad Nasir, Siti Munirah Mohd Faudzi, Noorfatimah Yahaya, Nor Suhaila Mohamad Hanapi, Wan Nazihah Wan Ibrahim

**Affiliations:** 1 Department of Chemistry, Faculty of Science, Universiti Putra Malaysia (UPM), Serdang 43400, Selangor, Malaysia; msnajihahnasir2496@gmail.com (N.M.N.); sitimunirah@upm.edu.my (S.M.M.F.); 2 Natural Medicines and Product Research Laboratory (NaturMeds), Institute of Bioscience (IBS), Universiti Putra Malaysia (UPM), Serdang 43400, Selangor, Malaysia; 3 Integrative Medicine Cluster, Advanced Medical and Dental Institute (AMDI), Universiti Sains Malaysia, Bertam, Kepala Batas, Penang 13200, Malaysia; noorfatimah@usm.my; 4 Faculty of Applied Sciences, Universiti Teknologi MARA, Shah Alam 40450, Selangor, Malaysia; norsuhaila979@salam.uitm.edu.my (N.S.M.H.); wannazihah@salam.uitm.edu.my (W.N.W.I.)

**Keywords:** natural products, molecular imprinted solid-phase extraction, polymerization technique

## Abstract

Molecularly imprinted polymers (MIPs) are synthetic polymers with a predetermined selectivity for a particular analyte or group of structurally related compounds, making them ideal materials for separation processes. Hence, in sample preparation, MIPs are chosen as an excellent material to provide selectivity. Moreover, its use in solid-phase extraction, also referred to as molecular imprinted solid phase extraction (MISPE), is well regarded. In recent years, many papers have been published addressing the utilization of MIPs or MISPE as sorbents in natural product applications, such as synthesis. This review describes the synthesis and characterization of MIPs as a tool in natural product applications.

## 1. Introduction

Natural products provide an infinite source for new therapeutic leads, for a treatment of a wide spectrum of disease. Historically, plants have formed the basis of traditional medicine system as documented in Mesopotamia (dating around ~2600 BCE), Egyptian medicine (dated from ~2900 BCE), “Ebers Papyrus” (dated from ~1500 BCE), Chinese Materia Medica (firstly dated in ~1100 BCE), Indian Ayurvedic system (dated from <1000 BCE) and by the Greeks and Romans, who’s contributed to the rational development of herbal-based drugs in Western world [1]. Pharmaceutical research expanded after the World War II (WW2) due to the discovery of penicillin, and by 1990, approximately 80% of drugs were either natural products or natural-based synthetic analogs were reported [2]. Some pertinent examples of plant-derived drugs are khellin, isolated from *Ammi visnaga* (L.) Lamk., which led to the development of bronchodilator agent, chromolyn (in the form of sodium chromoglycate) [3]. Plant-based system have continued to play an important role in healthcare since then, with the urgent needs for the searching of new pharmaceutics to treat cancer, HIV, and infectious diseases. Besides plants, marine organism and microorganism also significantly contributed towards the intensive investigation of nature as a source of novel bioactive agents. To name a few, halichondrin B, marine-derived anticancer agents, was derived from a complex polyether of several sponge sources [4], and bryostatin 1, which was isolated from complex macrolide extracts of the bryozoan, *Bugula neritina* [5,6]. Meanwhile, microorganism offers important antibacterial agents such as penicillins (from *Penicillium* species), and cephalosporins (from *Cephalosporium acremonium*) [7].

Following the review by Newman and Cragg [8,9] major categories of natural-based source of new active substances (NASs) or new chemical entities (NCEs) as proved by the FDA, dating from January 1981 through 30th of September 2019, including:

“B”: Biological; usually a large (>50 residues) peptide or protein either isolated from an organism/cell line or produced by biotechnological means in a surrogate host
“N”: Natural product
“NB”: Natural product “Botanical” (in general these have been recognized as drug entities by the FDA)
“ND”: Derived from a natural product and is usually a semisynthetic modification
“S*”: Made by total synthesis, but the pharmacophore is/was from a natural product

A prevailing sentiment in many pharmaceutical organizations is that the search for new bioactive compounds from the natural resources is hampered by the complexity of the work on the natural product extracts, in which the isolation and characterization of bioactive metabolites are challenging [10].

Since the most often used analytical procedures are costly and time-consuming, determining the most efficient methodologies and strategies is essential to achieving optimum results and cost-effectiveness. Molecularly imprinted polymers (MIPs) are one of the most recently recognized methods for characterizing new types of materials. The identification and separation of various natural compounds is one of the many applications of MIPs. MIPs are stable polymers with specific cavities designed for a particular template molecule, and possesses high selectivity compared to conventional solid phase extraction methods.

**Figure 1:**
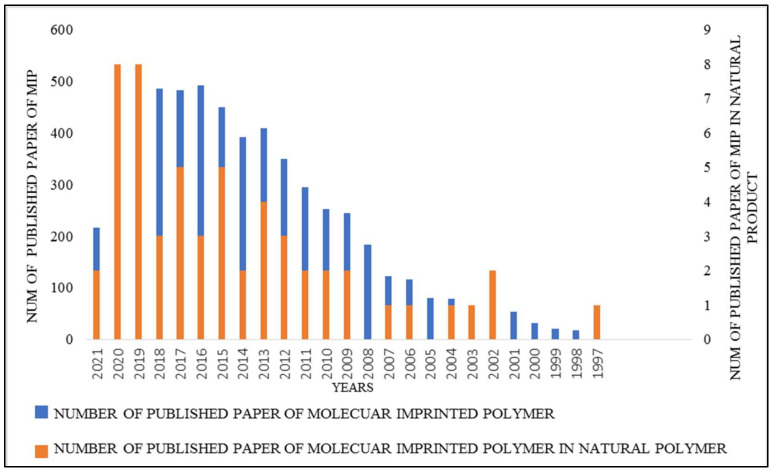
Number of published papers of MIPs and MIPs in natural product found in PubMed.

MIPs are synthetic materials that selectively rebinds a targeted molecule over other closely related compounds via its recognition site. Functional monomers and cross linkers are used to prepare MIPs by polymerizing around a template molecule, resulting in a three-dimensional network polymer with a high cross-link density. In this regard, the interaction ability of the monomer with the template molecule’s functional groups are taken into consideration. Vasapollo et al. [11] describes the mechanism in which the template molecule is removed after polymerization, and subsequently, binding sites with similar shape, size, and functionality to the target analyte are generated. This results in an imprinted polymer that is stable, robust, and resistant to a wide range of pH, solvents and temperature. Thus, MIPs behave similarly to natural receptor interactions in which a target molecule (i.e., antibody-antigen) is selectively retained, minus the associated stability limitations. Research in the field of MIPs has gotten much attention, as shown in Figure 1, due to their advantages such as their convenient and straightforward preparation, predetermined selectivity, and robustness in organic solvents—acidic or basic reagents, and durability in high temperatures.

To prepare MIPs, a functional monomer and crosslinker in the presence of a template are utilized. The template-monomer system is chosen so that the imprint molecule is able to forms a complex in solution with numerous functional monomers. The resulting imprints have steric (size and shape) and chemical (spatial arrangement of complementary functionality) memory of the template. These imprints enable the polymer to selectively rebind the imprint molecule from a mixture after removing the imprint molecules. In this regard, MIPs with template recognition site of natural products are also a useful method for identifying and extracting natural compounds. MIP-related research in the field of natural products has increased in recent years, as shown in Figure 1. Some examples of MIP applications in the field of natural material analysis have been described below.

Xie et al. [12] synthesized MIPs Artemisinin to determine its presence in *Artemisia annua* L. Artemisinin is used as an antimalarial drug, and its polymer demonstrates reliable recognition and selective ability after a series of adsorption experiments. Selective artemisinin recognition was achieved from the *A. annua* L. samples, implying its efficacy for determining artemisinin levels. Bhawani et al. [13] reported that gallic acid was extracted in fruits like blueberries, strawberries, apples, and bananas, as well as other plants and herbs. Gallic acid is a naturally occurring polyphenolic compound. Precipitation polymerization was utilized to synthesize molecularly imprinted gallic acid polymers for the tracing of gallic acid in urine samples, and the method was found to be reliable. The extraction efficiency of this method was tested by spiking samples with a standard gallic acid solution to observe how well it worked, whereby around 80% of gallic acid was extracted from the spiked urine sample.

## 2. Synthesis and Characterization of MIP

### 2.1. Polymerization Parameter in MIP Synthesis

The properties and performance of MIPs are influenced by a variety of factors. Each of the factors must be carefully chosen and optimised in order to obtain the best MIPs with the most favorable and highest of efficiencies. The influence of these parameters on MIP performance is discussed in this section.

#### 2.1.1. Functional Monomers

The selection of monomers is a significant step in the synthesis of MIPs. In 2016, Xiao et al. [14] proposed the selection of MIPs via UV-Vis spectra. In both non-covalent and covalent interactions, a monomer should be capable of bonding with a template. However, non-covalent interactions are the most common procedure for the synthesis of MIPs due to the lower intensity of the interactions involved [15]. Non-covalent is based on interaction between template and monomer through weak forces such as Van der Waals forces. This resulted facile template removal. Moreover, non-covalent interaction is simple and effective. However, it’s easy disrupt during template-monomer complex. Covalent is a slower binding which more tedious during template removal. Acrylic acid (AA), 2- or 4-vinylpyridine (2- or 4-VP), and methacrylic acid (MAA) are the most commonly used functional monomers. MAA is often used as a monomer due to its ability to interact with the template via both non-covalent and covalent interactions [16].

Furthermore, the presence of a carboxylic acid functional group acts as a hydrogen bond, and proton donors ensure that MAAs are widely used in the preparation of MIPs. Furthermore, when the chemical/compound used is present in acidic, basic, or neutral conditions, MAA creates hydrogen-bonding interaction with them. As such, an effective recognition component can be obtained by forming a potent complex between the functional monomer and template, of which is designed to complement the template. When the template contains primary functional groups, acidic monomers are preferred, for example, MAA. As the template contains acidic functional groups, a basic monomer such as 4-VP is preferred. 2-Hydroxyethyl methacrylate (HEMA) [17] is an example of an uncharged monomer. To achieve good selectivity, acrylamide (AM) could be used. When AM is added to a prepolymerization mixture, for example, it produces materials with high selectivity towards carboxylic [18], amide [19], and alcohol groups [20,21,22,23].

MAA and 4-VP are used in a study by Nunez et al. [24] to formulate the molecularly imprinted solid-phase extraction (MISPE) procedure in 2010. This is because non-steroidal anti-inflammatory drugs (NSAIDs) can bond to the hydrogen atoms of the basic amine (-NH) functional group when the carboxylic group is neutral, resulting in high extraction efficiency. When it comes to NSAIDs, it mainly uses 2-VP and 4-VP as monomers [25]. In contrast, MAA and other polar monomers cannot create hydrogen bonds because it is inefficient at interacting with weakly polar or polar templates. Although hydrophobic monomers like styrene can be utilized in these situations, designing and synthesizing monomers with complementary structures to the template structure that interact via hydrophobic and Van der Waals forces is preferable [26,27].

#### 2.1.2. Template Molecule

The selection of template molecules is based on several variables such as its stability, cost, solubility, toxicity, and functional groups that can interact with monomers [28]. MIP selectivity is greatly influenced by functional groupings, whereby its molecular recognition is based on the pre-organization and shape selectivity of the functional group. Previous studies have recommended the generation of binding sites that possess better specificity and affinity with numerous functional group interactions [29]. Simon et al. [30] demonstrated the remarkable selectivity of templates with two or fewer functional groups, indicating that shape selectivity is the most important mechanism for molecular recognition. This might be due to a conflict between shape selectivity and functional group pre-organization during imprinting, or a rebinding behavior that did not appear to be executed. The imprinting performance of the templates with three or more functional groups significantly improves with distance between the functional groups. Spivak et al. [31] discovered two notable observations towards MIP selectivity that were influenced by cavity shape. To begin with, steric limitations occur when a molecular structure that is too big cannot fit into an imprinted site that was produced using a minimal template molecule. In addition, to maximize Van der Waals interactions inside the MIP binding sites, the selected molecular structures must be equivalent to or smaller than the template molecule. Furthermore, diverse branching topologies outperform straight-chain hydrocarbons in terms of selectivity, with straight-chain groups possessing eight or more carbons completely losing recognition.

Researchers have explored the potential of increasing the template concentration while maintaining the monomer concentration, however, this approach does not increase MIP performance [32]. One of the reported adverse effects of introducing too many templates is the loss of site credibility [33]. When the template/monomer ratio improves, high-selective two-point binding sites should be replaced by a point binding site. Similar findings were subsequently achieved for templates with a single functional group that were anticipated to interact with the functional monomer once. The finding shows that the pre-polymer complex solution phase does not significantly influence the number of functional groups at the binding site of the polymer. Multi-functional binding sites result in the assembly of binding sites of polar functional groups, which occur during polymerization in the phase separation processes. Thus, the high-affinity binding sites of the imprinted polymer which is non-covalent are independent towards the pre-polymer stoichiometry due to the interactions between several monomers. In most applications, a starting working concentration of about 5% of the total monomer is utilized, then changed according to the experimental parameters [34].

To enhance recognition, the imprinting molecule should be structurally similar to the analyte [35]. While the target analytes appear to be a good fit for the template, numerous investigations have demonstrated that completely removing the template is challenging [36]. As a result, there are fewer accessible binding sites, but the remaining template is also released slowly, especially during trace analysis, ultimately influencing the final findings [37]. The usage of a dummy template (a template having a similar structure to the target analyte) potentially prevents problems caused by template leakage. Aside from that, a preliminary amount of the template (typically 1 mmol) must be present for MISPE preparation in a normal scale polymerization [28]. Ya et al. [38] and Osman et al. [39] reported that 1 mmol of template was used during preparation of MIPs is sufficient to ensure the formation of the defined sites with polymers. However, the selection on the concentration of the template usually depending on the overall ratio and scale of MIP production. If the analyte/template molecule is costly and complicated to prepare, a dummy template can be employed as a structural counterpart to replace it [40]. However, it must first be proven that the signal of the dummy molecule does not impede any measurements [37]. The leakage should then be reduced to tolerable levels by minimizing solvent variations, drying the sorbent, or performing thermal post-treatments [41].

MIP sensors used fluorescence and potentiometric methods to detect the presence of cocaine, according to BelBruno [42]. The former used a MIP made by combining a divinylbenzene functional monomer with EGDMA, as well as PEG-coated, Mn-doped ZnS quantum dots (QDs). The MIP particles were made on a solid substrate, and histamine was templated into a control polymer instead of the more common NIP or nonimprinted polymer. In comparison to the usual technique, real samples of human blood serum were assessed and determined to be accurately analysed. The successful use of a “dummy template”, a chemical replacement for the target of interest, was a distinctive aspect of this MIP.

#### 2.1.3. Crosslinker

Monomers are copolymerized around a template via covalent bonding, and are arranged in space to form stable complementarity. The majority of the imprinting methods require a high number of crosslinkers in order to recognize the functional monomers [41]. On the other hand, a crosslinker with more than 80% has a deleterious effect towards MIP performance. This might be due to the polymer that ends up blocking the binding sites, and furthermore, binding sites that are still accessible have a hard time removing templates, which in turn makes rebinding challenging [43]. However, the increase in crosslinker-solvent interactions results in mobile binding sites that reduces MIP recognition specificity, while the solvation propensity could be determined from the cross-performance linkers. *N*,*N*-methylenebisacrylamide (BisAA) and pentaerythritol triacrylate (PTA), for example, are superior to crosslinkers such as ethylene glycol dimethacrylate (EDMA) and divinylbenzene (DVB). PTA and BisAA solvate a lot of the popular solvents being used in synthesis and rebinding research. As there are fewer crosslinkers available to interact with, the general affinity of the MIPs become lower [44].

However, in order for the template rebinding with the MIP to occur, a minimum of wettability is required. Unlike EDMA, DVB is too rigid and inadequately solvated to provide adequate recognition properties. DVB is also less thermally stable than EDMA, making it the most effective crosslinker for various template molecules [29,45]. According to Gavrilovic et al. [46], “shape selectivity” can be improved if traditional polymerization reagents are added with a functional crosslinker. A functional crosslinker was synthesized based on the steroidal template, which was 5-dihydrotestosterone, 17-hydroxy-5-androstan-3-one (DHT), of which also possess chemical interactions (Van der Waals stacking interactions) with the template in the pre-polymerization mixture. As a result, the synthesized polymer increases the number of binding sites and improves their homogeneity, making the use of such functional crosslinkers very remarkable.

According to Mueller [47], the ratio of functional monomer to crosslinker is particularly important in this work because it controls the physical properties of the resulting MIP. Porogens are commonly used to improve the surface area of imprinted polymers and hence their capacity. One of the most important criteria in determining the surface area is the solubility of the template, monomer, and crosslinker. When phase separation causes the complex or polymer to precipitate, the surface area of the complex or polymer is reduced. If the non-solvent forms an emulsion, it can cause cracks or pores, increasing the surface area. Using a solid porogen, such as a salt particle that can be dissolved and washed off, is an effective technique to enhance surface area. The surface area, pore size, and binding capacity of the resultant polymer, as well as the structure of the pre-polymerization complex, are all affected by the crosslinker’s characteristics. The development of the pre-polymerization complex is driven by intermolecular forces between the template, monomer, crosslinker, and solvent, as proven by various computational investigations. As with porogens, this can result in phase separation, resulting in denser structures and reduced surface area. Because the strength of binding with molecules with similar qualities can be identical, this typically results in a variety of structurally distinct pre-polymerization complexes.

#### 2.1.4. Porogenic Solvent

All of the polymerization components are contained co-solvent, including the template, functional monomer(s) and initiator. Solvents plays a supporting role in the creation of macroporous polymer pores. Porogen was developed to describe the solvent for this reason. Depending on the type and concentration of the porogen, the shape and total pore volume of the macroporous polymers can be varied. To produce polymers with a well-developed pore structure and high specific surface areas, a thermodynamically favorable solvent is used. A thermodynamically ineffective solvent, on the other hand, produces polymers with poorly formed pore structures and low specific surface areas. During the polymerization step, the solvent also influences the time it takes for the developing polymer phase to separate. According to Guyot and Bartholin [48], greater solubility phase porogens would be preferable for applications in liquid conditions, such as solid phase extraction (SPE), since larger surface areas enhance cavity accessibility. However, as only a minimal volume of solvent is required, the polymer has a low surface area, making it thick and impenetrable [49]. Sellergren et al. [50] observed that using dichloromethane to increase selectivity resulted in a polymer with low porosity and a small surface area.

According to a research by Spivak et al. [35], optimal results are usually obtained with little or no porosity in a chloroform-based polymer. This demonstrates that there is no direct link between material identification properties and polymer topology. Solvation shows that porosity is not required for substrate diffusion through MIP. Furthermore, the polarity of the solvent is important as a solvent with a stable polarity is crucial for monomer-template assembly. Water is a favored solvent in situations when the template and monomers interact via hydrophobic interactions [51]. In addition, polar solvents, such as alcohol-water combinations, can imprint hydrophilic and weakly polar templates [52,53].

#### 2.1.5. Initiator

Heat, light, and chemical/electrochemical reactions are utilized to initiate free-radical polymerization in the presence of templates. Nonetheless, the investigation system may allow drivers to choose between the two. Photochemical or thermally triggered initiators, for example, would not be triggered if the template was photochemically or thermally unstable. When hydrogen bonding influences complexation, lower polymerization temperatures are preferred, and photochemically active initiators may be used in these cases due to their efficiency at low temperatures [51].

MIPs has a presence of specific recognition regions in the polymer net formed through template-tailored synthesis hence, MIPs are characterized by their selectivity and specificity. There are three stages to this synthetic process. First, the pre-polymerization structure or complex is formed from functional monomers and the templated molecule in the presence of an appropriate solvent. It is a crucial stage, since it determines the formation of the specific recognition regions responsible for the imprinted materials selectivity. In the case of MIPs, this step can be completed either by the covalent or non-covalent approaches, which differ in the type of interactions formed between the template and monomers. The covalent approach is executed via the formation of chemical bonds and, as a result, a new pre-polymerization compound is obtained. In the non-covalently formed pre-polymerization complex, weak molecular interactions. The following step of the imprinting process comprises of a polymerization reaction, usually in the presence of a cross-linker, to fix the pre-polymerization structure and form a polymeric matrix with the specific recognition regions and the template inside of them. In the last stage, the template is removed from the polymer by physicochemical processes, such as hydrolysis reaction (in the covalent approach) or desorption (in the non-covalent approach), yielding a highly cross-linked polymeric matrix with three-dimensional specific recognition regions complementary in term of the molecular volume and geometry of chemical functionalities to the imprinted entity. Those regions are able to adsorb/desorb the template molecule [54].

### 2.2. Polymerization Technique

Various techniques are used to prepare MIPs for this purpose, including precipitation polymerization, bulk polymerization, suspension polymerization, and in situ polymerization. Each of these techniques have their advantages and disadvantages, of which are described in this section.

#### 2.2.1. Bulk Polymerization

Copolymerizing functional monomers and crosslinkers commonly produce MIPs in the presence of a template molecule. However, the polymer produced may be irregularly shaped due to the crushing and grinding mechanisms involved, resulting in the destruction of the imprinted sites and cavities, low adsorption capacity, and poor site accessibility to the template molecule. At the same time, polymers of varying sizes are unfavorable for chromatographic applications.

**Figure 2:**
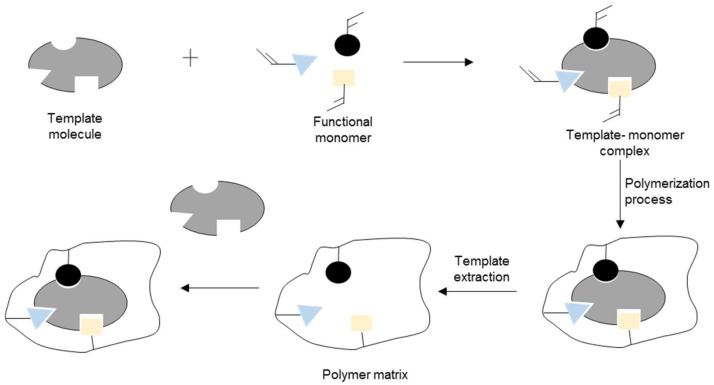
General Scheme for in situ polymerization [11].

During the mechanical disintegration of a synthesized polymer, the binding group may be partially destroyed, resulting in homogenous absorption sites. The approach is also characterized by low selectivity and reproducibility [55]. The synthesis apparatus used in this process is quite simple, and the reaction conditions are easy to control. Unfortunately, due to their varying sizes and shapes, the chromatographic performance of these particles is usually inadequate [56]. The general scheme for bulk polymerization is shown in Figure 2.

**Figure 3:**
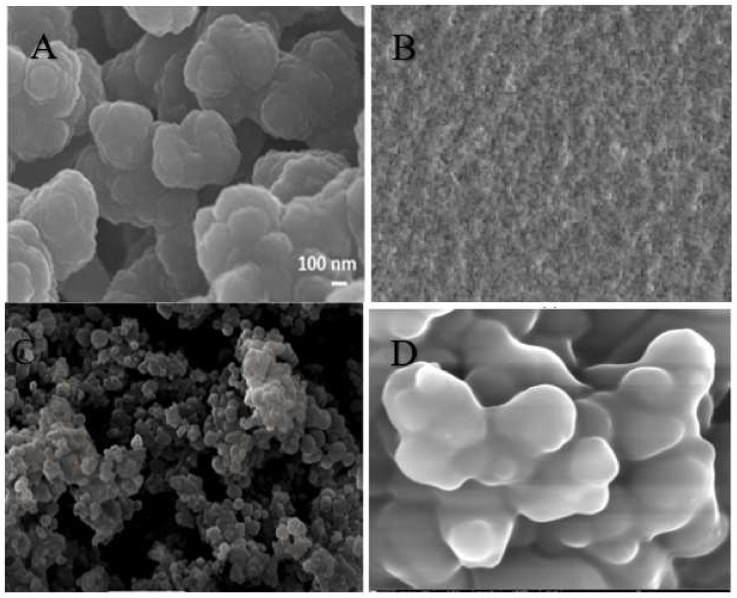
Scanning electron micrographs images of MIP; (**A**): bulk polymerization [58], (**B**): in situ polymerization [59]], (C): precipitation polymerization [60], (**D**): suspension polymerization [61].

As an example, Mohajeri et al. [57] compared the recognition of clozapine MIP prepared by bulk and the precipitation polymerization methods. Using clozapine as the template, MAA was chosen as a monomer due to the presence of the amine group in clozapine. The template easily bound with the acidic monomer, and EDMA was used as a crosslinker. The polymer synthesized via the precipitation technique was obtained without crushing and sieving, while the synthesis by bulk polymerization involved crushing and sieving. It was suggested that the binding sites of the polymer of the precipitation technique was more effective, whereby the particles obtained were spherical and more uniformly sized than bulk polymerization. Figure 3A shows scanning electron microscopy (SEM) images of a polymer that utilized the bulk polymerization technique.

Inexpensive and straightforward are several factors that make bulk polymerization a commonly used technique. Its one drawback is that the technique is time-consuming specifically during the polymerization process, involving tedious processes such as crushing, grinding, and screening. These techniques affect the polymer’s spatial structure and recognition sites, and also the polymer’s selective adsorption of the template molecules [40]. Furthermore, increasing the concentration of the crosslinking agent causes the template molecule to attach thoroughly; thus, an extra amount of eluent is needed to remove the template. Due to the low MIP yield produced with this technology, the implementation of this method in the industrial manufacturing and analytical laboratory sectors is impossible. To address these limitations, many polymerization techniques for the direct production of MIP spherical particles of the desired size have been developed.

#### 2.2.2. In Situ Polymerization

In situ polymerization is a particularly effective technique for the production of MIPs for high-performance liquid chromatography (HPLC) or solid phase extraction (SPE) separation. In a typical method, the reaction mixture, including the functional monomers, template molecules, porogenic agents, crosslinking agents, and initiators is placed in a stainless-steel tube, sealed at one end, and ultrasonically degassed. The process is then allowed to proceed by heating the other end to initiate polymerization. Once the template molecules have been removed, the MIP monolith column can be directly connected to the HPLC system for online SPE analyte quantification [55]. The general scheme for the in situ polymerization is shown in Figure 2.

The rod-shaped MIP separation medium is synthesized by direct polymerization in an empty column or capillary via in situ polymerization. The mixed solution for pre-polymerization is directly injected into a column or capillary to develop a continuous rod-shaped polymer. It provides consistency and homogeneity properties, as well as the capacity to provide excellent separation efficiency [55].

In situ molecularly imprinted SPE of matrine from radix *Sophorae tonkinensis* was developed by Guo et al. [62] in 2011. Matrine was made in the lab using melamine-urea-formaldehyde (MUF) as the functional monomer and matrine as the template in this study. The surface binding sites of the MIP could selectively recognize matrine when the template/functional monomer ratio (T/M) was 5 mg g^−1^. The MIPs had a high adsorption and elution ability towards the target molecule, indicating its excellent adsorption and elution ability. The SEM images of the polymer synthesized using the in situ polymerization technique is illustrated in Figure 3B.

#### 2.2.3. Precipitation Polymerization

**Figure 4:**
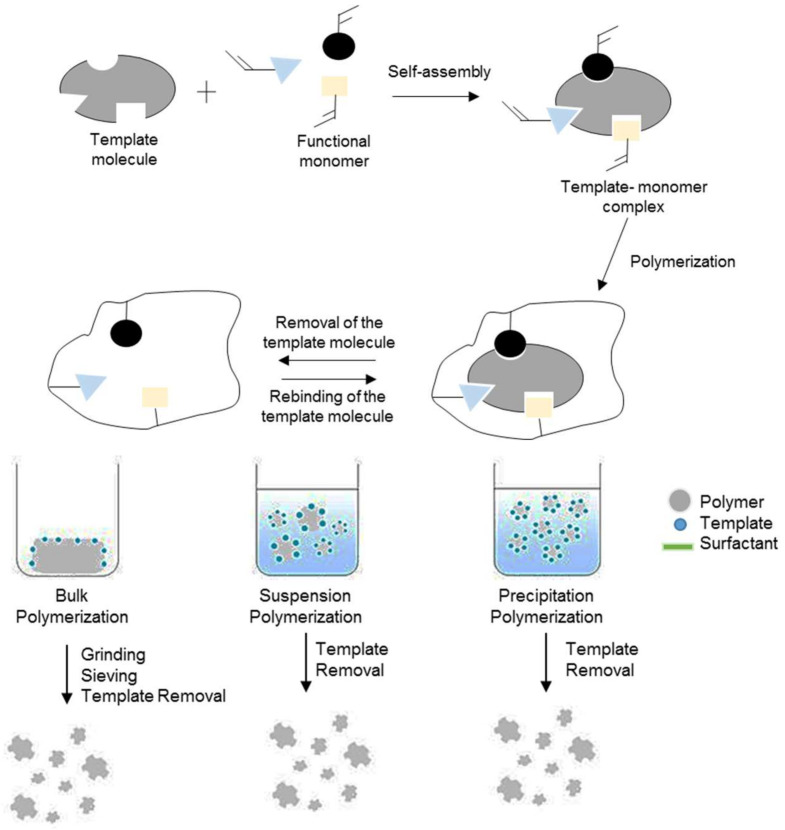
General scheme of polymerization techniques used for the preparation of MIP [63,64].

Precipitation polymerization is another direct and straightforward method for the synthesis of MIP beads, which are typically nanometer-sized and allow for the synthesis of uniform spherical particles (diameters typically less than 1 μm). However, it requires a significant amount of templates. Precipitation polymerization is a high yield polymerization process that requires one preparative step and is a surfactant-free process that involves polymerizing monomers in dilute solutions (without overlap or coalescence) and the precipitation of the polymer particles. The entropic precipitation of nanogel (seed) particles, followed by a continuous capture of oligomers from solution, is the most common way for the particles to proliferate. Compared to other polymerization techniques, such as bulk polymerization, this approach requires a high amount of solvents. It should also be highlighted that multiple parameter, including the polarity of the solvent, reaction temperature, and stirring speed, influence the size of the particles formed. Therefore, reaction conditions should be carefully monitored [55]. The general scheme for the precipitation polymerization technique is shown in Figure 4.

In precipitation polymerization, the monomer and initiator can be dissolved in the reaction medium, however, in the reaction mixture, the polymer is insoluble. Therefore, the polymer will precipitate out of the reaction system when a solid granular product has been formed. Precipitation polymerization is an efficient technique for synthesizing imprinted polymer microparticles with desirable properties regularly. The SEM images of the polymer synthesized using the precipitation polymerization technique is illustrated in Figure 3C.

Bhawani et al. [13] studied molecularly imprinted gallic acid polymers produced by precipitation polymerization. The interaction between the template and monomer was achieved using a non-covalent method throughout the synthesis process. Gallic acid was used as a template and acrylic acid as a functional monomer in the polymerization process. Bhawani et al. produced microspheres with smooth, clean surfaces and appropriate particle sizes for their investigation. The study revealed that the procedure was straightforward and has several advantages over the suspension polymerization technique because stabilizers are not used during the polymerization process. Furthermore, the precipitation polymerization technique contains no suspending agent or dispersion, overcoming the problem of the polymerization system’s high viscosity. When compared to MIPs made via suspension polymerization, the resulting polymers have high purity.

#### 2.2.4. Suspension Polymerization

Suspension polymerization is an efficient technique (one-step polymerization) and one of the most systematic approaches for creating MIP beads, allowing for the formation of spherical particles with a large size (in the range from micrometers to millimeters). This technique can produce high-quality MIP beads in the size range of 5 µm to 50 µm. However, the utilization of water is unsuitable with the non-covalent imprinting methods. In addition, the use of liquid fluorocarbons has its drawbacks, including being a generally expensive process [55]. The general scheme for the suspension polymerization technique is shown in Figure 4.

Suspension polymerization refers to the polymerization of monomers suspended in small droplets of water. It has two immiscible phases: continuous and dispersed. Functional monomers, initiators, porogens, and template molecules make up the dispersed phase. Polyvinyl alcohols are frequently used as a suspending agent in the continuous phase to improve their stability. Because a small droplet is comparable to a small unit from bulk polymerization, the shape and particle size of the polymer microspheres can be easily controlled. The porosity of the surface of the particles can be effectively adjusted by varying the amount of porogen. A suspending agent must be introduced to the solution to establish a protective film on the surface of the particles to prevent the particles from adhering to each other [56].

Polydatin was chosen as a template polyphenol by Gomes et al. [61] because of its large size and amphiphilic nature. The product obtained by a suspension polymerization technique, which included 4-VP as a functional monomer, EGDMA as a crosslinker, water/methanol as a solvent, n-heptane as an oil, and Span 80 as a surfactant, exceeded all other MIPs in terms of polyphenol uptake. This MIP was anticipated to have a maximum retention capability of q_max_ ∼300 µmol/g, which was substantially higher than the sorption capabilities described in the literature for comparable systems. The likely surface imprinting process caused by polydatin’s amphiphilic characteristics was the factor for such a significant result. These interfacial processes are likely to be responsible for the superior performance found with this suspension product compared to precipitation particles. The SEM images of the polymer synthesized using the suspension polymerization technique is illustrated in Figure 3D.

Contrarily, traditional techniques such as bulk, precipitation, in situ, and suspension technique uses large amounts of organic solvents. Water could potentially be a suitable alternative, owing to it generally being able to develop strong interactions with the template and/or monomers. However, water can destabilize the complex and interfere with the synthesis of the specific sites. Many of the limitations of the traditional MIP production processes can be solved by using green alternative solvents. Supercritical carbon dioxide (scCO_2_) is a green solvent that can potentially be used in polymerization techniques instead of typical organic solvents. The MIP will possess features including controlled morphology and homogeneous binding sites. Polymer synthesis in scCO_2_ has a number of advantages over conventional organic solvents, including the fact that it is aprotic and has high mass transfer and diffusivity, which is difficult to achieve with organic solvents. The ability to obtain polymers simply through depressurization, low toxicity, and low preparation costs, make scCO_2_ an appealing solvent to replace commonly used organic solvents. Presently, most commercially available monomers, including those often used in MIP synthesis, are soluble in scCO_2_. Compared to conventional bulk methods whereby the MIPs must be ground and sieved before use, resulting in irregular particles and destruction, MIPs developed using this green solvent are typically obtained as dry free-flowing powder which are pure, sterile, high in yield, and possess a narrow particle size distribution. These advantageous features the need for any further drying or purification steps, making the process ready to be used, and easy to handle [65].

According to Serpa et al. [66] to ensure higher greenness for the analytical methods there are several principles, such as: use multi-analyte methodologies over methods using one analyte at a time; and use reagents obtained from renewable sources. In fact, the majority of the current green analytical chemistry principles focus on sample preparation where direct analysis avoiding sampling; use of minimal sample size; perform in-situ measurements; and selection of automated and miniaturized methods. Moreover, solid-phase extraction (SPE) face criticism from a green chemistry perspective due to excessive use of hazardous organic solvents in multistep and laborious procedures. This is the reason why sample preparation is considered as the least green step of an analytical method. MIPs have not been unaware of the green materials synthesis trend. The introduction of several modifications on their synthesis have permitted a decrease in the consumption of organic solvents or their substitution by greener alternatives such as Ionic Liquids (ILs) and deep eutectic solvents (DESs), which can act as porogens, monomers, or even as crosslinkers. Besides, both solvents (Ionic Liquids and deep eutectic solvents) have been also used to improve the green features in other synthesis approaches for other material.

The reusability and stability of the imprinted material play a significant role in producing an economic, dependable, sustainable, and ecologically friendly strategy, according to Orowitz et al. [67]. Polymer degradation compounds are well-known for contaminating samples during their application. The crosslinker, crosslinking degree, condition template extraction, and functional monomer are the four primary parameters that influence MIP reusability and stability. DVB-based polymers exhibited an excellent outcome in terms of crosslinking degree, with DVB-based polymers being able to be reused at least 100 times without losing their template affinity under acidic and basic environments and at elevated temperatures. In contrast to DVB-based polymers, acrylamide and methacrylate-based polymers’ crosslinking degree reduced in both acidic and basic conditions due to irreversible breakdown. In molecularly imprinted microspheres (MIMs) preparation, both acrylamide and methacrylate crosslinkers are routinely utilized. Future research on establishing a long-term stable and reusable MIM formulation could face this difficulty. Alternative approaches that can be considered, according to these studies, include the use of green templates, green monomers, green solvents such as porogens and template removal solvents, green crosslinkers and initiators, energy efficiency, the use of ultrasound and microwaves to promote reaction rates, miniaturized techniques, and the use of computational tools for optimizing both the polymer and synthesis process. Higher energy demands have substantial environmental consequences, such as global warming.

When compared to conventional heating methods, Sajid et al. [68] stated that using comparatively greener energy sources such as microwave and ultrasound for extraction can reduce the impact on the environment and the analyst. Kalinowaski et al. [69] proposed a solution based on the green analytical chemistry principle: large volumes of analytical waste should be avoided, and proper management of analytical waste should be provided, which includes optimising extraction and clean-up stages using mathematical and statistical modelling.

Because of the great selectivity, sufficient clean-up, and enrichment abilities of the imprinted layer, Sobiech et al. [70] stated that combining nanostructures of Quantum Dots (QDs) or Carbon Dots (CDs) with MIP creates materials capable of selective adsorption of analytes from environmental, biomedical, or food samples. Because of their appealing optical characteristics, QD-MIPs materials are in high demand as detectors. CD-MIPs, on the other hand, have sufficient biocompatibility and low toxicity, allowing for targeted cell treatment and tissue imaging. However, there are a few flaws that must be solved at this time. To begin with, QDs’ high toxicity renders them exceedingly harmful to the environment. Novel QD nanomaterials based on ternary semiconductors or Perovskite materials may be a viable alternative in this case. Second, CDs with a low Quantum Yield (QY) may be limited in their practical application. To eliminate surface defects and increase QY of those nanoparticles, fresh synthetic techniques for CD manufacture must be devised.

All the discussed polymerization techniques applied in the synthesis of MIPs were summarized in Table 1.

## 3. Characterization of Molecularly Imprinted Polymer

Several authors have studied the application of MIP in natural products. For example, SEM is one of the most common methods for studying the porous structure and shape of MIPs in images. Moreover, Brunauer-Emmett-Teller (BET) are used to studies on the surface area through the adsorption of N_2_ is the most common method for assessing the surface area and morphology of porous solids. As a result, porous materials with a wide surface area can be employed to immobilize and adsorb analytes. Furthermore, their stability can be determined using thermogravimetric analysis (TGA) and Fourier-transform infrared (FT-IR).

Bi et al. [86] reported the synthesis of MIPs using an anion-exchange mechanism to minimize non-directional ion-ion interactions acids from a natural plant extract of phenolics while reducing other interfering substances that could decrease selectivity during anion exchange. The FT-IR spectra of IAP and IMAP indicated peaks at 1634 cm^−1^, which are expected of the imidazolium groups, and at 1730 cm^−1^, which are representative of EDGMA’s C=O group. Between 3075 and 3090 cm^−1^, no significant band indicative of C=CH_2_ was found. These results show that the polymers were successfully prepared. SEM and BET were used to analyze the structures of P9 and P18. The monolithic structures were most likely formed from their comparable monolithic column synthesis. Furthermore, P9 and P18 showed sub-porous structures when magnified to 600 nm, and a BET investigation determined their surface areas of 50.7 m^2^ g^−1^ and 72.4 m^2^ g^−1^, respectively, with average pore sizes of 50.2 Å and 45.6 Å, which were attributed to cavity formation by molecular imprinting, effectively increasing the surface area of the ionic liquid-based IMAP as the average pore size decreased.

The in situ polymerization reaction was used to synthesize molecularly imprinted silica monolithic (MISM), as reported by Arabi et al. [87]. To produce a crack-free and non-fragile structure, tetraethyl orthosilicate (TEOS), 3-aminopropyl trimethoxysilane (APTMS), gallic acid, and thiourea were utilized as crosslinker, functionalized monomer, template, and precursor, respectively. After removing the template, the FT-IR spectra of the molecularly imprinted silica and non-imprinted silica monolithic were analyzed. Due to the sol-gels’ high water adsorption capacity, a large absorption band at 3300 cm^−1^ was determined, of which was related to OH stretching and represented the adsorbed water on/into the monolithic silica structure. At 783 and 1456 cm^−1^, weak absorption asymmetric stretching bands of C=S were observed. The absorption band at 475 corresponded to the N=C=N rocking mode. The silicate groups (Si–O) overlapping with the C=S stretching vibrations contributed to the band at 1089 cm^−1^. The peak for the N–H group was identified at approximately 3369 cm^−1^, while the peak for the Si-OH group was observed at approximately 970 cm^−1^. The primary band spectra of the imprinted monolithic and non-imprinted monolithic had the same position and appearance due to the removal of the template from the sol-gel structure.

Under optimum preparation conditions, the morphology of MISM was characterized by SEM, which showed that it possesses a porous and permeable structure with a large pore size. Several macro-pores and flow-through channels suggests that the material was suitable for use as an SPE adsorbent. Thus, the enormous surface area and high adsorption capacity of a homogeneous macro-pore structure could theoretically be achieved.

**Figure 5:**
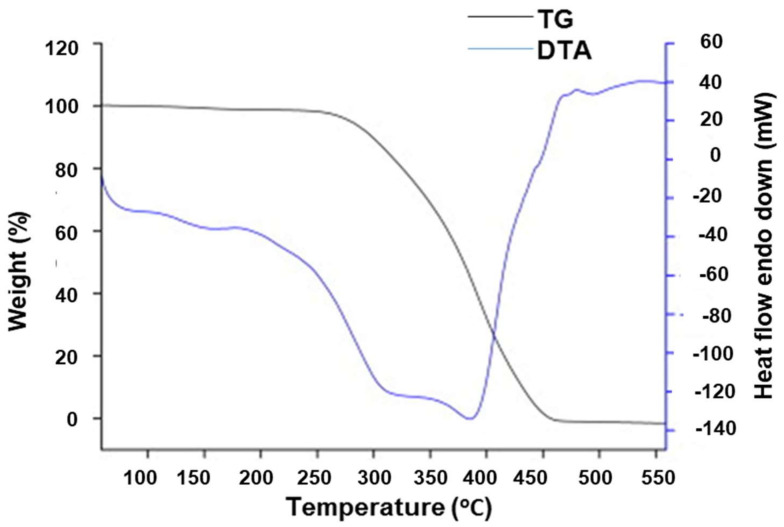
Thermogravimetric analysis (TG) and differential thermal analysis (DTA) of hypericin-imprinted polymer [77].

Li et al. [77] studied MIPs prepared via an oxidation-reduction polymerization system using a non-covalent molecularly imprinting strategy with hypericin as the template, acrylamide as the functional monomer, and pentaerythritol triacrylate as the crosslinker in an acetone porogen. TG and DTA (Figure 5) were used to analyze the mechanism of decomposition of a described hypericin-imprinted polymer (P1-M) in a dynamic nitrogen atmosphere. The decomposition temperature was 277.68 °C, and the mass-loss ratio was 99.88% when the temperature reached 455.97 °C, according to the TG plot. The DTA curve shows that the melting point temperature and enthalpy of melting (△H) were 387.02 °C and 6717.36 J g^−1^, respectively, at the starting temperature (228.48 °C) of the thermal absorption.

**Figure 6:**
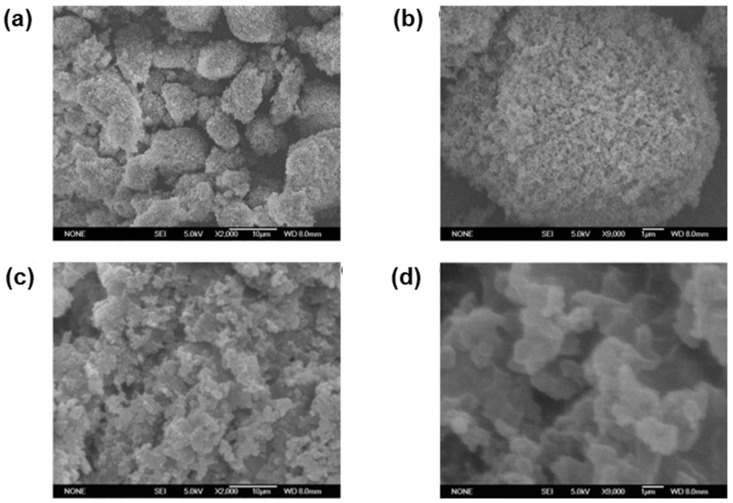
SEM images of hypericin-based (**a**,**b**) MIP and (**c**,**d**) NIP [77].

SEM images of the surface morphologies of MIPs and NIPs are shown in Figure 6. The MIP particles (Figure 6a,b) were more porous, had bigger pore sizes, and had a rougher structure than the NIP (Figure 6c,d). It can be concluded that MIPs with a more uniform and open structure is significantly better for template embedding and mass transfer than NIPs.

A more detailed discussion of the relationship between the pore system of different bulk imprinted polymers and their sorption properties was described by Granados et al. [88] researched synthetic methods for the selective extraction of hydroxylated metabolites from human urine samples. In this study, it was observed that the specific surface area of MIP was higher than of NIP. The fact was explained by the MIP had six times the pore volume of the equivalent NIP. In addition, studies conducted by Samah et al. [75] says that pore volume of NIP somewhat lower than MIP. On the contrary, Cantarella et al. [89] studies shows that specific surface area of NIP was higher than of MIP. As, the NIP had a larger total pore volume (0.51 cm^3^/g) compared to the MIP (0.39 cm^3^/g) in conjunction with the higher SSA. The interaction of the DCF molecules as a template with MAA, which could partially inhibit crosslinking, potentially explains the MIP’s lower pore volume compared to the NIP. These findings support the notion that the imprinting effect, and not the pore size distribution, is responsible for the MIP’s adsorption effectiveness.

**Figure 7:**
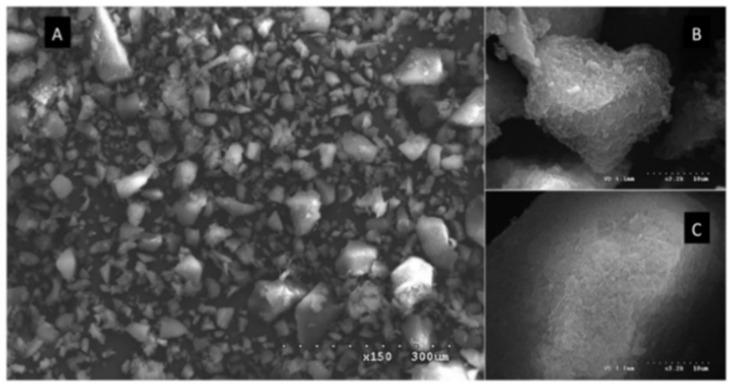
SEM microscopic images of crushed polymers produced by the bulk polymerization method. (**A**,**B**) shows the MIP particles, while (**C**) shows the NIP particles [74].

The morphology of MIP and NIP particles by Granados et al. [88] showed in Figure 7A shows SEM images of MIP particles made from crushed polymer monoliths generated by bulk polymerization, of which possess a non-spherical shape (A). On the imprinted Figure 7B and non-imprinted surfaces Figure 7C, the textural surface of the polymers exhibits a typical pattern (C). The NIP, on the other hand, possesses a distinct roughness and a smoother surface. Similarly with Huang et al. [90] that investigated the use of propyl gallate (PrG) as a template in a MIP cartridge. Shows that the morphology of MIP displayed a rougher and more porous surface compared to NIP. Sanagi et al. [91] also reported that the surface morphology and image of the MIP had rough surface with an irregular order. As the pore sizes of the samples ranged from 2 to 50 nm, the polymers were classified as mesoporous.

The morphology of MIPs that characterized by SEM via the suspension technique was discuss detailed by Liu et al. [85] to synthesis MIPs for the selective extraction of 4-hydroxybenzoic acid (4-HB). It shows that the MIP particles were spherical, with sizes ranging from 1 to 8 μm, with a peak value of 5 μm, while the MIP microspheres have an average diameter of 4.8 μm. On the other hand, the NIP microspheres have an average size of 4.5 μm and a peak value of 5 μm. The MIP microspheres have a rougher surface structure than the NIP microspheres. In addition, the MIP’s uniform and accessible structure makes it more favorable towards 4-HB adsorption. Similarly, as He et al. [92] shows that the MIPMs and NIPMs were spherical and possess a smooth surface with particle sizes in the μm range. The MIPM particles, which had an average particle diameter of 150 μm, were smaller than the NIPM particles with 250 μm in diameter.

In BET characterization, He et al. [92] shows that the MIP had a specific area of 283.92 m^2^ g^−1^, whereas the NIP had a specific area of 253.34 m^2^ g^−1^. The template’s binding force with the function monomer may have forced the MIPMs to be tighter and smaller, resulting in a more significant specific area and tremendous adsorption advantage over the NIPMs. The surface of the MIPMs had denser and more uniform micropores than the NIPMs. Furthermore, the presence of a template was thought to have an impact on the polymer morphology. As such, the MIPMs’ uniform and open structure made it easier to remove and embed the target molecule compared to NIPMs.

**Figure 8:**
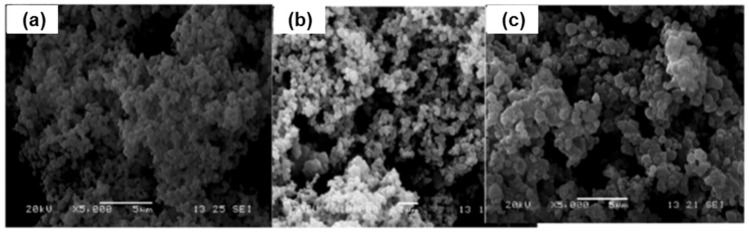
SEM images of MIPAM with various crosslinkers: (**a**) 3:1; (**b**) 4:1; (**c**) 5:1 [60].

In a precipitation polymerization, the morphology was discussed by Li et al. [93]. The SEM analysis shows that spherical particles were obtained with a diameter of around 0.2 μm for the MIPs, while an interconnected-irregular bulk was observed in the NIPs. This morphological characterization shows the successful synthesis of MIPs and further proves by Lu et al. [60] revealed that MIP particles were spherical and well dispersed. The morphology of MIP_AM_ synthesized at various crosslinkers is illustrated in Figure 8. The particle size of the MIP (M8) was small at crosslinker = 3 (Figure 8a), and it failed to form stable spheres, as the crosslinker increases from 4:1 to 5:1 [Figure 8c], the MIP particles gradually disperse and form microspheres (M9) with relatively consistent diameters of ~2.1 μm. (M6). MIPs using different monomers showed a similar pattern.

**Figure 9:**
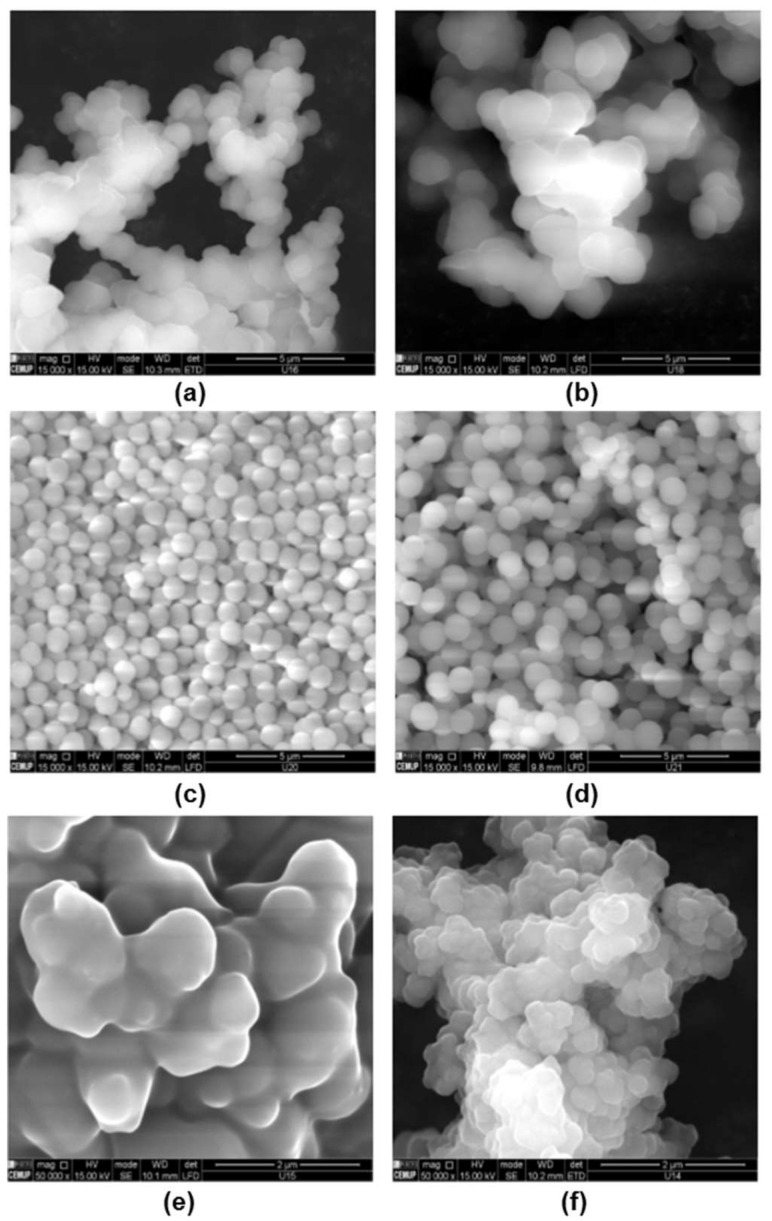
SEM images for different MIPs synthesized in this research: (**a**) MIP1 (prec. MAA/ACN/MeOH); (**b**) MIP3 (prec. AAm/ACN/MeOH); (**c**) MIP5 (prec. 4VP/MeOH/H2O); (**d**) MIP6 (susp. 4VP/DMF); (**e**) MIP7 (susp. 4VP/MeOH/H2O); (**f**) MIP8 (prec. AA/ACN/MeOH) [61].

Besides that, SEM micrographs for some of the polymer obtained from Gomes et al. [61] study are shown in Figure 9. This illustrating the influence of the synthesis conditions on MIP morphology. MIP1 and MIP2 (MAA and 4VP as functional monomers, respectively, both with ACN/MeOH as solvent) produced particles with a diameter less than 1 μm. The latter appeared to have a higher agglomeration. MIP3 (AAm as a functional monomer in ACN/MeOH) was used to generate aggregates consisting of particles with a particle diameter of less than ~2 µm, and a mild agglomeration phenomenon was observed. Due to the influence of the solvent utilized (Toluene/MeOH) with 4VP as the functional monomer, MIP4 has a considerably distinct morphology (nearly plain surface at the μm scale). Individual particles with a particle diameter of less than ~1 µm were produced without agglomeration effects using 4VP as the functional monomer and MeOH/H_2_O as the solvent (MIP5). In the monomer phase, suspension polymerization with 4VP as the functional monomer and DMF as the solvent resulted in comparable particles (MIP6). MIP7 should have had more potent agglomeration effects due to the suspension polymerization using 4VP as functional monomer (FM) but using MeOH/H_2_O as the solvent. Other imprinting techniques, as represented by MIP8 (AA as a functional monomer in ACN/MeOH and TMPTA as crosslinker), have similarly produced aggregates consisting of particles with a diameter of less than 1 µm, indicating some degree of product agglomeration. It is worth noting that, in addition to the concentration effect on particle agglomeration, other factors such as phase segregation thermodynamics and even stirring conditions might influence the development of such aggregates.

Gomes et al. [61] other reaction systems show interesting characteristics with regards to the impact of polymerization conditions on product morphologies. For example, MIP9 (DMAEMA as FM) has a significantly larger agglomeration than MIP8, indicating that the FM has a significant impact on product morphology, even at low concentrations (65%). On the other hand, the SEM micrograph of NIP1, generated under comparable conditions to MIP1 but without polydatin, indicates the production of individual particles with a smaller diameter and no agglomeration effects. As a result, a significant influence of the template molecule on particle production is observed. The morphologies that correspond to EGDMA and MAA particles, formed under MIP1/NIP1 conditions, show the entropic and enthalpic precipitation mechanisms involved in producing such polymer structures. Indeed, in some types of polymerization processes, such as MAA polymerization in ACN/MeOH, the developing polymer chains might phase-separate from the continuous medium by precipitation due to unfavorable enthalpic polymer-solvent interactions. Precipitation can also be caused by an entropic effect caused by crosslinking, which inhibits the solvent and polymer from mixing, as in EGDMA polymerization. However, in many circumstances, such as the creation of MIPs/NIPs, both entropic and enthalpic precipitation mechanisms may be implicated, with simultaneous impacts on the morphology of the products.

**Figure 10:**
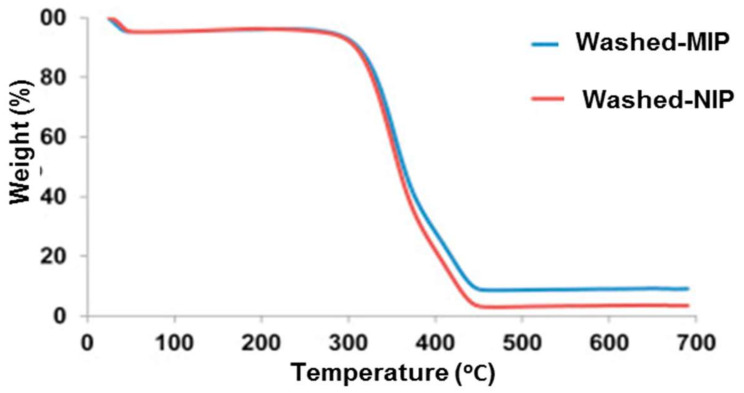
Thermogravimetric analysis of the synthesized polymers [94].

The stability of polymer of bulk polymerization method characterized by TGA was discuss by Cantarella et al. [89] to synthesis of MIPs by remove drugs from water. In this study, TG and DTG curves of the MIP indicate the beginning of decomposition at 211 °C and the maximum degradation rate temperature (MRDT) at ~390 °C, with a shoulder placed at approximately 300 °C. The residue level in the MIP sample was low, whereas the degradation at approximately 82 °C indicates residual CH3CN. The NIP’s TG and DTG curves were also observed and compared with the MIP. The thermal profile showed a slightly higher decomposition temperature (~400 °C) compared to the other shoulders due to the various decomposition stages. The MIP’s MRDT reading suggests that DCF molecules influenced the polymerization reaction, resulting in a lower molecular weight (MW). From this, it can reasonably be assumed that a decrease in MW causes a shift in the thermal profile towards lower temperatures, resulting in shoulders overlapping. Apart from that, Zunngu et al. [94] produced a selective MIP for ketoprofen shows the decomposition of MIP and NIP at ~290 °C (see Figure 10) designated as the temperature at which the polymer backbone collapses. The polymers lost about 4% of their mass when heated to 40 °C, with the adsorbed methanol utilized in the template removal stage most likely the cause of this loss.

For characterization by FTIR was discuss by Sanagi et al. [91], shows that the C=O stretching vibration absorbance in the range of 1700–1750 cm^−1^ was indicated to crosslinking polymerization of EGDMA and MAA. The O–H band was visible as absorption at 3600–3400 cm^−1^, whereby the MIP intensity before washing was lower than after washing, although its intensity was identical to that of the NIP’s. This behavior could be explained by the fact that the template molecule (quinalphos) bonded to the monomer (MAA) via hydrogen bonding with the hydroxyl group during the synthesis of MIP prior to washing. Due to the lack of hydrogen bond breaking, a high and broad stretching vibration absorbance peak of the hydroxyl group from the monomer was easily recognizable when the template was removed. The washing stage successfully leached the template out, as shown by the decrease in intensity.

**Figure 11:**
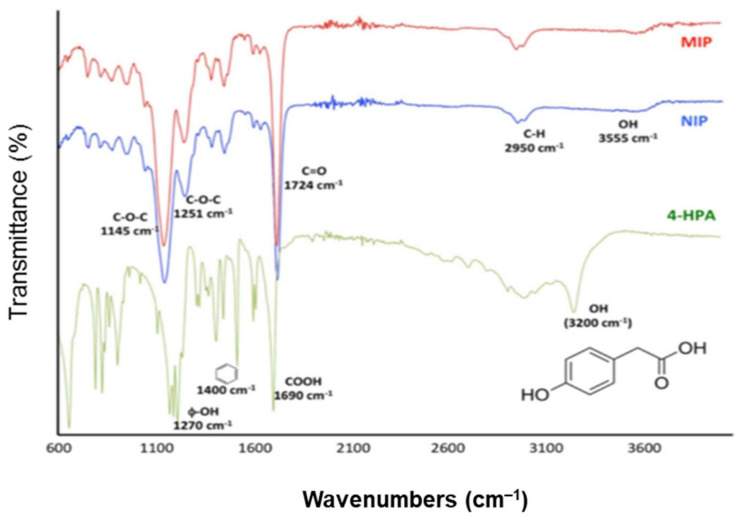
ATR-FTIR spectra for MIP, NIP, and 4-HPA [74].

Granados et al. [88] Figure 11 shows the MIP, NIP, and 4-HPA template spectra generated using an attenuated total reflection-FT-IR (ATR-FTIR). The polymerization state can be determined from the ATR-FTIR analysis. The absence of bands in the range 1680–1640 cm^−1^ confirms the polymers’ complete polymerization by indicating the absence of vinyl groups in the polymers. 4-HPA has distinct bands at 3200 and 1690 cm^−1^, both of which correspond to carboxylic acid O–H and C=O stretching, respectively, while 1400 cm^−1^ relates to benzene ring vibrations and 1270 cm^−1^ attributed to phenol O-H stretching. A successful extraction process was also indicated by the absence of distinctive bands for the template. The intensity of the C=O stretching vibration band was represented at 1724 cm^−1^ in the current spectra.

Liu et al. also characterized the FT-IR spectra of the MIP and NIPs of interest [85]. The polymer network’s C–H symmetric and asymmetric stretching of methyl and methylene groups was attributed to the doublet peaks at 2957 and 2990 cm^−1^ of the MIP and NIP in the IR spectra. The carbonyl groups of the crosslink agent EGDMA were responsible for the strong peaks at 1726, 1724, and 1160 cm^−1^. The C=N and C=C stretching vibrations of the pyridine units caused by the 4-VP monomer in MIP and NIP, respectively, were attributed to the adsorption peak at 1636 and 1454 cm-1. However, the lack of adsorption bands at 2136 and 1948 cm^−1^ may be attributed to the 4-VP vinyl bending vibrations, suggesting that the 4-VP was involved in the polymerization process.

He at al. [92] successful synthesis of imprinting sites was observed using an FT-IR spectroscopy. Several absorbance peaks of the aromatic ring group band at 1600–1450 cm^−1^ was shown after template removal, including the C=C stretching vibrations of the phenol in DES at 1514 cm^−1^, indicating the successful synthesis of DES imprinting sites. After the DES removal, the aromatic ring group band was removed in the MIPMs, indicating that the template molecule was washed away entirely by the Soxhlet extraction. Between the MIPMs and NIPMs, there was not much of a distinction. The stretching vibration of –OH was assigned to the peak around 3568 cm^−1^, while the stretching vibrations of the saturated and unsaturated alkyl groups had two peaks at 3000 cm^−1^. The stretching vibration of the –C=O was implicated for the new absorbance peak at 1735 cm^−1^. A band at 1637 cm^−1^ was attributed to the C=C group stretching vibration. The stretching vibrations of C–O in carboxylic acid and ester were observed to be around 1261 cm-1 and 1159 cm^−1^, respectively, indicating the presence of MAA and EGDMA in the polymer polymerization. Furthermore, due to hydrogen’s presence between DES and MAA, the peak intensity in the NIPMs was lower than that in the MIPMs.

Li et al. [93] observed absorptions in the azoxystrobin molecule of the MIPs correlated to the stretching vibration of –CN on the aromatic ring and the plane bending vibration of –CH on the aromatic ring at 2230 and 1560 cm^−1^, respectively, while the carboxyl groups in MAA were assigned at 1731 cm^−1^. Two distinct peaks of MIPs at 2230 cm^−1^ and 1560 cm^−1^ disappeared after washing with HAc–MeOH, suggesting that the azoxystrobin template was removed. These findings validated the presence of templates and monomers in the polymers and ultimately a successful polymerization was achieved.

**Figure 12:**
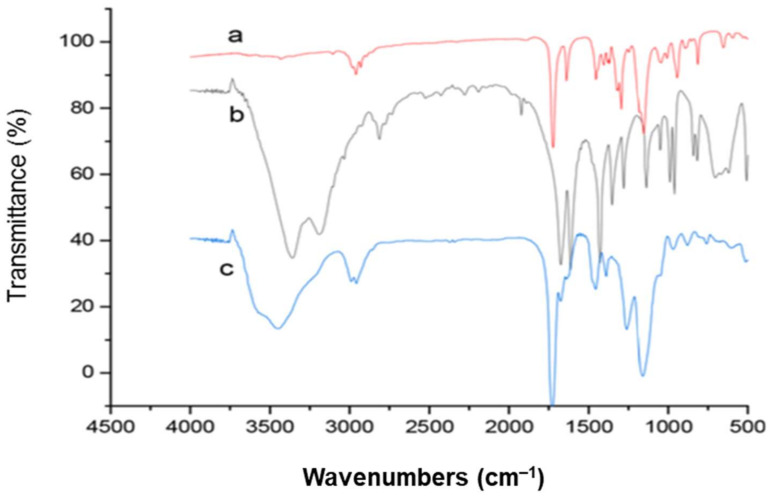
FT-IR spectra: (**a**) EGDMA; (**b**) AM; (**c**) MIPAM [52].

Lu et al. [60] observed peaks (Figure 12) at 1150 cm^−1^ may be related to the vibration of the C-O bond stretching in EGDMA (a). Peaks at 3400 cm^−1^ correspond to –NH groups, 1637 cm^−1^ to the stretching vibration of the C=C double bond, and 1720 cm^−1^ to the stretching vibration of C=O groups, respectively, in AM (b). The existence of EGDMA in MIPs was confirmed by the presence of two different peaks at 1720 cm^−1^ (C=O stretching) and 1150 cm^−1^ (C=O stretching) in MIP_AM_ (c). MIP peaks at 3400 cm^−1^ (NH stretching) shows that the functional monomer AM was successfully polymerized with EGDMA. The vital binding sites for template molecules will be of these amino groups.

**Figure 13:**
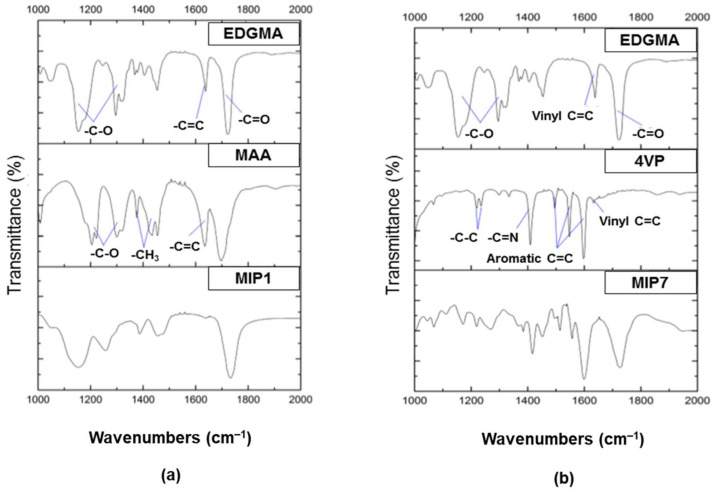
(**a**) FT-IR spectra collected for the MIP1 product (synthesized with MAA as functional monomer and EGDMA as crosslinker); (**b**) FT-IR spectra collected for the MIP7 product (synthesized with 4VP as functional monomer and EGDMA as crosslinker) [61].

Figure 13 shows two imprinting systems analyzed by Gomes et al. [61] as examples of this type of analysis (MIP1 and MIP7). Figure 13a,b also includes FT-IR spectra for the isolated and purified MIPs, as well as spectra for the constitutive functional monomers (MAA or 4VP) and crosslinker (EDGMA). As a minimal peak was detected for the final materials at approximately 1630 cm^−1^ (a well-known C=C assignment), it is reasonable to conclude that a very high rate of C=C bonds conversion occurred. Furthermore, the integration of the functional monomer in the final MIP (a significant concern in terms of molecular imprinting efficiency) was clearly demonstrated in these studies. Peak assignments that match the functional monomer (e.g., aromatic C=C in 4VP at ~1550 cm^−1^ or ~1600 cm^−l^) may be characterized in the synthesized MIPs, though they were not observed in the crosslinker.

## 4. Molecular Imprinted Polymer in Solid Phase Extraction (MISPE)

**Figure 14:**
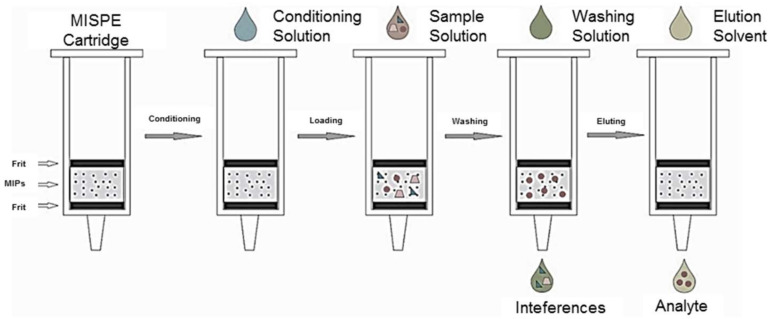
MISPE procedure [95].

There are four important steps in the MISPE, including (i) conditioning, (ii) loading, (iii) washing, and (iv) elution (Figure 14). The MIP sorbent is usually packed between the fritz in the SPE cartridge. The offline and online modes are the two most basic and widely used modes in SPE, and it is necessary to optimize a number of parameters in both modes. These parameters include contact time, sample strength, sample pH, amount of sorbent used, sample flow rate, the addition of salt and buffer solution as well as the washing solvent, the elution solvent, and the loading solvent [95].

### 4.1. Offline Modes

In recent years, offline MISPE has seen a rise in popularity. Offline MISPE operations are identical to traditional SPE processes consisting of four important steps (conditioning, sample loading, washing, and elution). The MIPs are packaged into cartridges using a standard process that contains 15–500 mg of MIPs [64].

Firstly, the cartridge is conditioned with a conditioning solvent in order to optimize interactions between the analyte’s target in the sample and the MIP [96]. Sample loading is one of the most essential steps of MISPE. In this step, the sample medium must be carefully chosen because it has a significant effect on the MIP’s recognition towards the analyte. The type of template/monomer interactions that occur during polymerization, as well as the porogen used, determines the loading solvent. To bind analytes at the imprinted locations, an electrostatic driving force, such as ionic interactions and hydrogen bonding, is typically used. Therefore, when loading samples, a low-polarity solvent should be used to establish the interaction towards analytes. Toluene, chloroform, acetonitrile, and dichloromethane have been reported as the most widely used solvents. However, it should be highlighted that when an aqueous solution containing the analyte of interest is saturated at the MIP surface, hydrophobic interactions might cause the analyte and other interfering chemicals to be retained non-selectively together with the analyte [55].

The washing step is utilized to maximize the particular interactions between the analytes and MIP while removing the interfering components that have been retained in the polymer matrix. The washing solvent should be selected based on the analyte, impurity, and the characteristics of the components. This step usually uses a low-polarity organic solvent such as dichloromethane, chloroform, toluene, or a mixture of these solvents. When electrostatic interactions drive recognition, the usage of porogens during polymerization, similar to the loading step, is a better option. However, using an aqueous solution on a MIP column is difficult since many porogens are incompatible with water. Thus, the MIP column should be nitrogen-dried prior to the washing stage. The polarity of the solvent, on the other hand, must be enhanced when non-specific interactions are needed. To improve washing selectivity, a small amount of polar components such as water, alcohol, acetonitrile, or base is frequently utilized. In this case, it is necessary to manage the washing solvent volume, additive, and pH [55].

The final step in MISPE is elution. To achieve significant enrichment factors, only a small amount of solvent is necessary. Protic/polar solvents such as acetonitrile or water, or a mixture of both are widely applied in the elution step. Weak acids (acetic acid or trifluoroacetic acid) or weak bases (triethylamine or pyridine) in small amounts may be used to achieve significant recoveries due to the strong interactions between the MIP and the analyte. Following elution, the eluate typically undergoes a re-constitution step (dried, followed by dissolving in a suitable solvent) to increase the extraction enrichment factor. For the quantification of the analyte, several detection methods such as chromatography, capillary electrophoresis (CE), electrochemical methods, and atomic absorption spectrometry (AAS) is used. However, the offline approach typically takes a lengthy amount of time, which potentially increases analytical mistakes [55].

Chen et al. 2012 [97] analyzed the compound Kirenol using the offline mode. Kirenol is a terpenoid derived from Siegesbeckiae, a Chinese herbal medicine that has been used to treat inflammatory, allergic, and arthritic conditions. Their MIPs and NIPs’ molecular selectivity was determined through the evaluation of the sorbents via offline MISPE protocols. The MISPE was synthesized with a mixture of solvents with different porogens and types of template-monomer interactions during polymerization. Kirenol is a polar molecule that does not have any hydrophobic functional groups. According to the findings, MIPs made in tetrahydrofuran showed stronger recognition capabilities than those prepared in *N,N*-dimethylform amide or acetonitrile:methanol (1:1). Different quantities of kirenol in solution were used to identify the optimal solvents (water, acetonitrile, and tetrahydrofuran), and were loaded in the MISPE column in the range of 0.5–2 mL. The eluate was collected, and an HPLC analysis was subsequently performed. The best solvent was found to be acetonitrile, which had an optimal volume of 1.5 mL. Furthermore, 5 mL of acetonitrile was chosen as the optimal volume for the washing step. The best elution solution was determined to be 5 mL of methanol/acetic acid (90:10, *v*/*v*). According to the findings, the spatial orientation of the functional groups in specific binding sites was a significant factor in non-covalent MIP molecular recognition. MISPE’s binding affinity for kirenol was highly selective, and it could potentially rebind other structurally related diterpenoids. However, binding affinity for diterpenoids was lower than kirenol.

### 4.2. Online Modes

**Figure 15:**
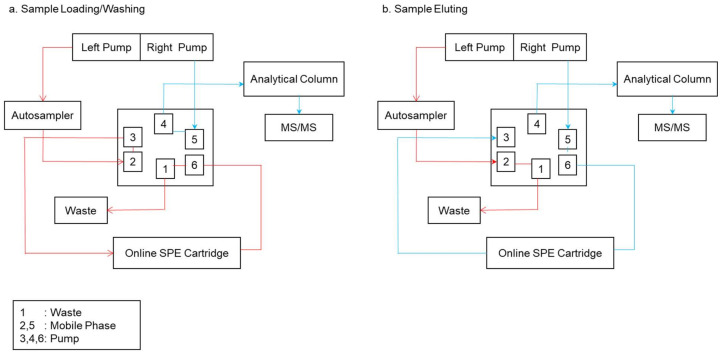
Molecular imprinted solid-phase extraction (MISPE) steps in online mode (**a**) Sample Loading/Washing; (**b**) Sample Eluting [95].

In contrast to the offline mode, as shown in Figure 15, the online mode provides for automatic sample loading, interference washing, analyte elution, separation, and detection by an analytical equipment. In a typical process, a tiny pre-column filled with molecularly imprinted polymers is placed in the loop of a six-port injection valve. The sample is placed into the analytical column, interferences are removed, and the analytes are eluted and separated in the analytical column before being evaluated by the detector [95]. The following is a schematic diagram of the MISPE steps in online mode:

In online MISPE mode, the left pump conducts the autosampler procedure. The sample is then discarded after passing through the online cartridge. The right pump inflates the mobile phase into the analytical column, which is subsequently examined by the instrument. The left pump works in tandem with the correct pump during the eluting process, as shown in Figure 15b. The difference between the loading and washing processes is that the left pump’s sample does not go through an online cartridge, instead it goes through the right pump’s mobile phase.

The left pump transports the mobile phase, while the auto sampler transports the sample and mobile phase to the loops of port injection valves 2 and 3, then into the online SPE cartridge (containing the analyte), and the solvent to the loop of port injection valve number 1, before the stream is diverted to waste (see Figure 15a). The washing solvent is then transported to ports 2 and 3 via the left pump, entering the online SPE cartridge to wash the sample in the online SPE cartridge (Figure 15a). Next, the interferences stream is discharged, but the analyte remains trapped in the online cartridge. Figure 15a depicts the optimal pump that is used in the eluting process. The right pump transports the eluting solvent through ports 5 and 4 before passing it through the analytical column to elute the analyte for mass spectrometry (MS/MS) analysis.

During the eluting stage, the correct pump in Figure 15b transports the eluting solvent into the online SPE cartridge through ports 5 and 6, which carries the analytes from the online cartridge to ports 3 and 4 for MS/MS analysis. Figure 15b also depicts the process of the analyte being trapped in the online SPE cartridge and the stream being diverted to waste early in the loading step for the left pump. If the mobile phase is inadequate to elute the analytes from the MIP pre-column, another eluting solvent can be utilized. The eluted analytes then pass through the injection loop before being injected into the chromatographic set-up, where the mobile phase carries them away [90,98].

The online method has certain significant advantages over the offline approach, including solvent conservation and low cost. In some situations, the analytes are collected in a minimal solvent volume after loading the full sample extract through the MIP’s bed, resulting in a superior concentration factor. The MISPE online mode is most suited to multi-analyte determinations, where the MIP detects numerous structural analogues [90,99]. As a result, it is not as popular as the other modes.

As an example of analytes analyzed using the online SPE method, Bjarnason et al. [100] used simazine as a template in MIPs to extract triazines from humic acid (SPE). Online trace enrichment of water samples containing 20 ppm humic acid was proven to be successful using MIPs. Because of the MIP selectivity, large volumes of water samples containing humic acid (20 ppm) can be extracted with little influence from the matrix on the chromatogram, thus, high sample-enrichment factors could be obtained. On the other hand, sample matrixes like apple extracts or urine were retained on the MIP to some extent, limiting the amount of samples that could be extracted but still resulting in sample enrichment [100].

The high pressure of the online system when the column is loaded with nano-sized materials and the addition of an easily damaged SPE column are also downsides of the online mode approach. A strategy for controlling the drawbacks of column-based SPE has been proposed [95].

Over the last two decades, magnetic solid phase extraction (MSPE) methods based on magnetic adsorbents have become one of the most extensively used methods for separating and extracting organic, inorganic, and bioactive species at the matrix level. The MSPE method is based on the adsorption and desorption of analytes on magnetic adsorbents added to the analyte-containing sample solution. Magnetic particles are used to modify various types of polymers, nanomaterials, metals, and metal oxides that can be used as adsorbents in this process. Adsorbents that do not have magnetic characteristics are given magnetic properties in this method. During the process, the magnetic sorbent is added into the sample solution containing the analytes. In order to adsorb the analytes on the sorbent, the sorbent with the sample solution is allowed to interact for a certain period of time. The resulting mixture consists of the sample solution, and the magnetic sorbent is mixed for a certain amount of time in order to make the interaction more effective, faster, and simpler. The sorbent is isolated from the sample solution by an external magnetic field after the completion of the adsorption process. Next, an eluent is added to the sorbent for the elution of the target analytes, and the sorbent is isolated from the eluent phase by an external magnetic field. The concentration of target analytes in the eluent phase is then analyzed by a suitable detection system [101].

Li et al. in 2009 reported an important application of the online MSPE, whereby they fabricated a poly(dimethylsiloxane)(PDMS)/glass hybrid microchip for online SPE and electrophoresis separation of the trace amounts of fluorescence isothiocyanate (FITC)-labeled phenylalanine (Phe). The extraction phase was prepared by modifying the magnetic microspheres with hydroxyl-terminated poly-dimethylsiloxane (PDMS-OH), and conveniently immobilized into the SPE channel by a magnetic field. In this system, the injection of the sample solution into the SPE channel (PDMS-OH microspheres bed) and desorption of analyte from the sorbent phase into the electrophoresis channel was electrically driven [102].

According to Turiel et al. [103], molecularly imprinted solid-phase microextraction (MI-SPME) has become one of the most popular sample preparation techniques, and it is now widely employed in analytical laboratories. Incorporating molecular imprinting technology into the SPME appears to be a perfect way to offer selectivity to the extraction process while maintaining the SPME’s ease of operation and solvent less nature. During the last few years, there has been a lot of study into developing ways for preparing MIP-based fibres. These procedures have been aimed at obtaining two types of MIP fibres: MIP-coated fibres and MIP monoliths.

The detail of selected works in the area of MISPE were tabulated in Table 2.

## 5. Future Perspective and Conclusions

According to Fresco-cala et al. [113], molecularly imprinted polymers have recently become a perfect option for the selective and sensitive detection of target molecules in complex matrices where other structurally identical and related chemicals may coexist. Although MIPs exhibit the inherent qualities of polymers, such as stability, resilience, and ease/low cost of synthesis, when nanoparticles are introduced into their polymeric structure, certain of their characteristics can be increased, and new functions can be produced. The large number of nanoparticles accessible greatly increases the chances of finding the right nanostructured MIP design for any analytical challenge. Furthermore, depending on the synthesis method employed, different structures (such as monolithic solids or MIPs micro/nanoparticles) can be created.

Conventional SPE methods are typically burdened by the required additional solvents, possible evaporation and reconstitution steps, and additional time involved. Recently, in an attempt to address these limitations, a molecular imprinted solid-phase extraction has been demonstrated to be a reliable and cost-effective technique for the selective isolation and concentration of a wide range of analytes and sample matrices, and offers many improvements over traditional techniques, although there have been some limitations to the methodology, such as the amount of time required to perform any analysis. As there are already multiple measures to prevent or even minimize these limitations, MISPE is regarded as a robust analytical tool. It is worth noting that, while MIPs are difficult to optimize, their versatility and sensitivity make them ideal for identification and purification analysis.

There are many applications of MIPs in the various scientific fields. We are currently concentrating on their synthesis and characterization. Presently, the preparation of MIPs has been validated by a wide range of scientific experts that are interested in the development of MIPs that can be adapted into microextraction and solvent-free techniques, which bodes well for the future of this field of study. MIPs can be incorporated into contemporary sample preparation microextraction techniques without affecting their inherent selectivity and stability by making clever (and relatively simple) changes to the polymerization procedures. The developed methods for creating MIP-based microextraction devices are reliable and straightforward, and they can be carried out in any laboratory with the necessary equipment.

## Figures and Tables

**Table 1:** Various polymerization techniques for the synthesis of molecular imprinted polymers.

Type of Polymerization	Template Molecules	Functional Monomers	Crosslinkers	Initiator	Porogen Solvent	Washing Solvent	Ref.
Bulk	Clozapine	Methacrylic acid	Ethyleneglycol dimethacrylate	Azobisisobutyronitrile	Chloroform	Acetic acid/methanol	[57]
	Quercetin	Acrylamide	Ethyleneglycol dimethacrylate	Azobisisobutyronitrile	Tetrahydrofuran	Acetic acid/methanol	[20]
	Propranolol (Racemic)	Methacrylic acid	Ethyleneglycol dimethacrylate	Azobisisobutyronitrile	Toluene	Acetic acid/methanol	[71]
	Estradiol	4-Vinylpyridine	Ethyleneglycol dimethacrylate	Azobisisobutyronitrile	Acetonitrile	Methanol	[72]
	Serotonin	Methacrylic acid, 2-Hydroxyethylmethacrylate	Ethyleneglycol dimethacrylate	2 2’-azobis(2 4-dimethylvaleronitrile)	Acetic acid/methanol/Acetonitrile	Acetic acid/methanol/water	[58]
	2,4-Dinitrophenol	Acrylamide	Ethyleneglycol dimethacrylate	Benzoyl peroxide	Acetonitrile	Methanol/acetic acid	[73]
	Benzylparaben	Methacrylic acid	Trimethylolpropane trimethacrylate	Benzoyl peroxide	Toluene	Methanol/acetic acid	[74]
	Diclofenec	Allylthiourea	Ethyleneglycol dimethacrylate	Azobisisobutyronitrile	Acetonitrile	Methanol/acetic acid	[75]
	Kukoamine	Itaconic acid	Ethyleneglycol dimethacrylate	1, 1’-azobis (cyclohexanecarbonitrile)	Dimethylformamide	Methanol	[76]
	Hispidin	Acrylic acid, Methacrylic acid, 4-Vinylpyridine	Ethyleneglycol dimethacrylate	Azobisisobutyronitrile	Dimethyl sulphate, Tetrahydrofuran	Ethanol/Acetic acid	[77]
In situ	6-Amino caproic acid	Caprolatam	Graphene Oxide	Potassium persulfate	Sulfuric acid	Boiling water	[78]
	*S*-(-)-amlodipine	Acrylamide	Ethyleneglycol dimethacrylate	Azobisisobutyronitrile	Dodecanol	Methanol/Acetic acid	[79]
Precipitation	Clozapine	Methacrylic acid	Ethyleneglycol dimethacrylate	Azobisisobutyronitrile	Chloroform	Acetic acid/methanol	[57]
	Aristolochic acid	Acrylamide	Ethyleneglycol dimethacrylate	Azobisisobutyronitrile	Dimethylformamide	Methanol/Acetic acid	[14]
	Carbamazepine, Clofibric acid	2-Vinylpyridine	Ethyleneglycol dimethacrylate	Azobisisobutyronitrile	Toluene	Methanol/Acetic acid	[80]
	Diclofenac	2-Vinylpyridine	Ethyleneglycol diacrylate	Azobisisobutyronitrile	Toluene	Methanol/Acetic acid	[81]
	Rivastigmine	Acrylamide-2-methylpropanesulfonic acid	Ethyleneglycol dimethacrylate	Benzoyl peroxide	Acetonitrile	Methanol/Acetic acid	[82]
	Dibutylphthalate	Acrylamide	Ethyleneglycol dimethacrylate	Azobisisobutyronitrile	Acetonitrile	Ethanol	[83]
	Vitamin E (α-tocophenol)	Acrylamide	Ethyleneglycol dimethacrylate	Azobisisobutyronitrile	Acetone	Methanol/Acetic acid	[60]
	Gallic acid	Acrylic acid	Ethyleneglycol dimethacrylate	Azobisisobutyronitrile	Acetonitrile	Methanol	[13]
Suspension	Dibenzothiophene	4-Vinylpyridine	Ethyleneglycol dimethacrylate	Azobisisobutyronitrile	Toluene	Ethanol	[84]
	Polydatin	Acrylic acid, Methacrylic acid, Acrylamide	2-(Dimethylamino)ethyl methacrylate, *N*-vinylpyrrolidone, Ethyleneglycol dimethacrylate), Trimethylolpropane triacrylate	Azobisisobutyronitrile	Acetonitrile, Dimethylformamide, Methanol	*n*-heptane/span 80	[61]
	4-hydroxybenzoic acid	4-Vinylpyridine	Ethyleneglycol dimethacrylate	Azobisisobutyronitrile	Acetonitrile	Methanol/Acetic acid	[85]

**Table 2:** Selected works in the area of molecular imprinted solid phase extraction.

Template	Analyte	Monomer/Porogen Solvent/Cross linker/Initiator	Polymerization Technique	Template Removal	MISPE Mode	SPE Condition	Adsorption Evaluation	Ref.
Ketoprofen	Wastewater	2-VP/ACN: Toluene/ EDGMA/ ABCHC	Bulk	Acetic acid: ACN (1:9; *v*/*v*)	Offline	Cartridge: 14 mg Sample: 50 mL Conditioned: Methanol (1 mL), Deionized water (1 mL) Washing: 1 mL of 5% (*v*/*v*) triethiamine in water Eluted: Methanol (1 mL)	Comparing MIP-SPE efficiency with NIP-SPE	[94]
PMPA, EMPA, IMPA, CMPA, BMPA, MPA	Human Serum	MAA/ ACN/ TRIM/ ABCHC	Bulk	Methanol: Acetic acid (9:1; *v*/*v*)	Offline	Cartridge: 500 mg Sample: 1 mL Conditioned: 30% Hydrochloric acid (5 mL) Washing: Water, ACN Eluted: Water	The recoveries for degradation products is in range 81.2–90.5%	[104]
Monocrotophos	Water, Soil sample	MAA/ DCM/ EDGMA/ AIBN	Bulk	Methanol: 10% Acetic acid	Offline	Cartridge: 200 mg Sample: 1 mL Conditioned: Methanol (1 mL), LC Grade Water (2 mL) Washing: 2 mL CH_2_Cl_2_: ACN (95:5, *v*/*v*) Eluted: CH_2_Cl_2_: Methanol (90:10, *v*/*v*)	Extraction of 1 L river water at 100 mg/L spike level at range 77.5–99.1% while 5 g soil sample at 100 μg/kg at range 79.3–93.5%	[105]
Artemisinin	Artemisinin (Anti malaria drug)	Styrene/ ACN/ EDGMA/ AIBN	Bulk	Methanol: Acetic acid (9:1; *v*/*v*)	Offline	Cartridge: 100 mg Sample: 2 mL Conditioned: ACN (5 mL) Washing: Methanol: Acetic acid (9:1; *v*/*v*) Eluted: 5 mL Methanol: Acetic acid (9:1; *v*/*v*)	40 mg in 5 mL ACN solution: Q_MIP_: 8.46 mg g^−1^ Q_NIP_: 4.49 mg g^−1^	[12]
Quinalphos	Quinalphos (Organophosphorus pesticides)	MAA/ ACN/ EDGMA/ AIBN	Bulk	Methanol: Acetic acid (9:1; *v*/*v*)	Offline	Cartridge: 100 mg Sample: 10 mL Conditioned: Methanol (10 mL), Deionized water (10 mL) Washing: 5% Acetic acid in methanol Eluted: Methanol (6 mL)	High extraction recovery of analyte with MIPs compared to NIPs and C_18_	[91]
Florfenicol	Florfenicol	4-VP/ THF/ EDGMA/ AIBN	Precipitation	Methanol	Offline	Cartridge: 120 mg Sample: 10 mL Conditioned: Methanol (10%, *v*/*v*), Aceticacid (5%, *v*/*v*), water Washing: Aceticacid (1%, *v*/*v*) Eluted: ACN: water (25%, *v*/*v*)	120 mg in 10 mL in various concentrations shaken 3 h at RT: Q_MIP_: 4.32 mg g^−1^ Q_NIP_: 2.88 mg g^−1^	[106]
Diethylstilbestrol (DES)	DES	APTES/ Methanol/ TEOS/ Activated silica gel	Surface	Methanol: Hydro-Chloric acid (1:1; *v*/*v*)	Offline	Cartridge: 200 mg Sample: 1 mL Conditioned: Methanol (10 mL), Water (10 mL) Washing: 2 mL Methanol: Water (98:2; *v*/*v*) Eluted: 2 mL Methanol	50 mg in 10 mL in various concentrations shaken at 1 h at RT: Q_MIP_: 62.58 mg g^−1^ Q_NIP_: 19.89 mg g^−1^	[107]
Diethylstilbestrol (DES)	Seawater	MAA/ Chlorofoam/ EDGMA/ AIBN	Suspension	Methanol (5 mL), Water (5 mL)	Offline	Cartridge: 100 mg Sample: 20 mL Conditioned: Methanol (5 mL), Water (5 mL) Washing: 1 mL Methanol: Acetic acid (99:1; *v*/*v*) Eluted: 1 mL Methanol: ACN (65:35; *v*/*v*)	20 mg in 20 mL in methanol/water (60:40, *v*/*v*) shaken at 300 rpm: Q_MIP_: 8.43 mg g^−1^ Q_NIP_: 4.43 mg g^−1^	[92]
Catechol	Catechol	4-VP/ ACN/ EDGMA/ AIBN	Bulk	Methanol: Acetic acid (4:1; *v*/*v*)	Online	Cartridge: 70 mg Sample: 1.3 mL Conditioned: 2% ACN (*v*/*v*) Washing: Nitric acid, ACN Eluted: Nitric acid	Permanganate solution used reduced from Mn(VII) to Mn(II) at 528 nm	[108]
Kirenol	Kirenol	AA/ THF/ EDGMA/ AIBN	Bulk	Methanol: Acetic acid (9:1; *v*/*v*)	Online	Cartridge: 100 mg Sample: 6 mL Conditioned: ACN (5 mL) Washing: Methanol (5 mL), Acetic acid (5 mL)	Comparing MIP recognition ability with NIP.	[97]
Naproxen	Urine	4-VP/ Toluene/ EDGMA/ AIBN	Bulk	-	Offline	Cartridge: 200 mg Sample: 20 μL Conditioned: 6 mL ACN/Water/Acetic acid (60:30:10), 6 mL Mili Q Water (pH3) Washing: 2 mL ACN Eluted: 3 mL ACN/1% Acetic acid	Naproxen selectively extracted by MISPE	[99]
Simazine	Simazine	MAA/ DCM/ EDGMA/ AIBN	Bulk	-	Online	Sample: 200 μL Conditioned: 2 mL Water	Large volume sample extract and low detection limit acquired	[99]
4-Nitrophenol	Environmental water	4-VP/ ACN/ EDGMA/ AIBN	Bulk	-	Online	Sample: 20 μL Conditioned: 2 mL ACN, 2 mL Mili Q Water (pH 2.5) Washing: 0.4 mL DCM, 2 mL Mili Q Water (pH 2.5) Eluted: 1% CAN	Comparing efficiency of MIP-SPE and NIP-SPE	[109]
Sulfamethazine	Sulfamethazine	APTES/ ACN/ TEOS/ Activated silica gel	Surface	Methanol	Online	Cartridge: 50 mg Sample: 10 mL Conditioned: Methanol	Comparing efficiency of MIP-SPE and NIP-SPE	[110]
4-Hydroxylphenylacetic acid	Human Urine	4-VP/ Toluene/ EDGMA/ AIBN	Bulk	10% Acetic acid/ Methanol (*v*/*v*)	Offline	Cartridge: 100 mg Sample: 0.5 mL Conditioned: 2 mL Water, 2 mL ACN Washing: 1 mL Water Eluted: 1.5 mL ACN/1% formic acid	20 mg in 2.5 mL in solution incubated for 24 h: Q_MIP_ > Q_NIP_ IF > 3.5	[88]
Cephalexin	Human serum	TFMAA/ ACN/ EDGMA/ AIBN	Bulk	Methanol/20% Acetic acid	Online	Cartridge: 50 mg Sample: 20 μL Conditioned: ACN Washing: 1% TFA Methanol Eluted: Methanol (3 mL)	The detection limit was estimated at 0.04 μg mL^−1^ of cephalexin	[111]
Dextromethorphan	Human plasma	MAA/ Chlorofoam/ EDGMA/ AIBN	Precipitation	Methanol/ Phosphate Buffer	Online	Cartridge: 120 mg Sample: 50 μg^−1^ Conditioned: Methanol (1 mL), Ultrapure Water (1 mL) Washing: 0.1 M Hydrochloric acid (1 mL), Ultrapure Water (1 mL), DCM (1.5 mL) Eluted: 3 × 1 mL Methanol/Phosphate Buffer (90:10) (0.05 M, pH 5)	25 mg in 100 mL solution at shaken 30 min: Q_max_ MIP is 90 mg g^−1^	[112]
Theophylinne	Serum	MAA/ Chlorofoam/ EDGMA/ AIBN	Bulk	Methano/Acetic acid (9:1)	Online	-	Comparing efficiency of MIP-SPE and NIP-SPE	[71]
Endorine	Aqueous sample (river, tap water)	Provided by POIYINTELL	Surface	-	Offline	Cartridge: 100 mg Sample: 100 mL Conditioned: ACN (5 mL), Water (5 mL) Washing: 4 mL (water, ACN; 80:20), 2 mL water Eluted: Methanol (1 mL)	MISPE high recoveries than commercial C_18_ SPE	[24]

Pinacolyl methylphosphonate (PMPA, degradation product of Soman), ethyl methylphosphonate (EMPA, degradation product of VX), isopropyl methylphosphonate (IMPA, degradation product of Sarin), cyclohexyl methylphosphonate (CMPA, degradation product of GF), isobutyl methylphosphonate (BMPA, degradation product of a Russian VX) and methylphosphonic acid (MPA, the final degradation product of all nerve agents), Trimethylolpropane trimethacrylate (TRIM), 1,1-azobis(cyclohexanecarbonitrile) (ABCHC), methacrylic acid (MAA), 2-Vinylpyridine (2-VP), Dichloromethane (DCM), Tetraethoxysilicane (TEOS), 3-aminopropyltriethoxysilane (APTES), Azobisisobutyronitrile (AIBN), Ethylene glycol dimethacrylate (EGDMA), Methacrylic acid (MAA), 4-Vinylpyridine (4-VP), Tetrahydrofuran (THF), Acetonitrile (ACN).

## Data Availability

All data generated or analyzed during this study are included in this published article.

## Abbreviations

2-VP, 2-Vinylpyridine; 4-HB, 4-hydroxybenzoic acid; 4-VP, 4-vinylpyridine; AA, acrylic acid; ABCHC, 1,1-azobis(cyclohexanecarbonitrile); AIBN, 2,2′-azobisisobutyronitrile; AM, acrylamide; APTES, 3-aminopropyltriethoxysilane; APTMS, 3-aminopropyl trimethoxysilane; AT, allylthiourea; BET, Brunauer-Emmett-Teller; BisAA, *N*,*N*-methylenebisacrylamide; BMPA, isobutyl methylphosphonate; CH_3_CN/ACN, acetonitrile; CHCl_3_, chlorofoam; CMPA, cyclohexyl methylphosphonate; DCF, diclofenac; DCM, dichloromethane; DES, diethylstilbestrol; DHT, 5-dihydrotestosterone; DMAEA, 2-dimethylaminoethyl acrylate; DMF, dimethylformamide; DTA, differential thermal analysis; DTG, derivative thermogravimetry; DVB, divinylbenzene; EDMA, ethylene dimethacrylate; EGDMA, ethylene glycol dimethylacrylate; EMPA, ethyl methylphosphonate; FM, functional monomer; FTIR, fourier-transform infrared spectroscopy; H_2_O, water; HAc–MeOH, acetic acid-methanol; HEMA, 2-hydroxyethyl methacrylate; HPLC, high-performance liquid chromatography; IAP, IL anion-exchange polymers; IMAP, ionic liquid-based molecularly imprinted anion-exchange polymer; IMPA, isopropyl methylphosphonate; IR, infrared; MAA, methacrylic acid; MeOH, methanol; MIPMs, molecularly imprinted polymer microspheres; MISPE, molecular imprinted solid phase extraction; MMIP, magnetic molecularly imprinted polymer; MISM, molecularly imprinted silica monolithic; MIPs, molecularly imprinted polymers; MPA, methylphosphonic acid; MRDT, maximum rate decomposition temperature; MUF, melamine-urea-formaldehyde; Mw, molecular weight; NIPMs, nonmolecularly imprinted polymer microspheres; NIPs, nonimprinted polymers; NSAIDs, non-steroidal anti-inflammatory drugs; OPP, organophosphorus pesticides; PMPA, pinacolyl methylphosphonate; PrG, propyl gallate; PTA, pentaerythritol triacrylate; SEM, scanning electron microscopy; SPE, solid phase extraction; SSA, specific surface area; TEOS, tetraethoxysilicane; TEOS, tetraethyl orthosilicate; TG, thermogravimetric analysis; THF, tetrahydrofuran; TMPTA, trimethylolpropane triacrylate; TRIM, trimethylolpropane trimethacrylate.
